# Diffuse Alveolar Hemorrhage: A Rare Life-Threatening Condition in Systemic Lupus Erythematosus

**DOI:** 10.1155/2012/836017

**Published:** 2012-05-27

**Authors:** Ravi Paul Singh Virdi, Adeel Bashir, Ghulamullah Shahzad, Javed Iqbal, Jose O. Mejia

**Affiliations:** Department of Internal Medicine, Nassau University Medical Center, East Meadow, NY 11554, USA

## Abstract

Diffuse alveolar hemorrhage (DAH) is a rare life-threatening complication in systemic lupus erythematosus (SLE) associated with high mortality rates. DAH is more common in women, and mean age of onset is around 30 years. It mostly occurs in patients with established diagnosis of SLE but can be the initial presentation of lupus in approximately 20%. DAH should be suspected in lupus patient presenting with new pulmonary infiltrates, decline in hemoglobin, hemoptysis, dyspnea, hypoxemia, and increase in carbon monoxide diffusion capacity. Radiographic evidence of bilateral pulmonary alveolar infiltrates that are usually perihilar or basilar with sparing of apices is seen. DAH can often mimic clinically and radiologically severe pneumonia or ARDS. Treatment includes high-dose corticosteroids, cyclophosphamide, and plasmapheresis. We report a case of diffuse alveolar hemorrhage complicating SLE flare-up in a male patient.

## 1. Introduction

Pulmonary complications in systemic lupus erythematosus (SLE) can occur in 50–70% of affected individuals during the course of their illness [1–3]. The spectrum of pulmonary manifestations includes pleuritis, interstitial lung disease, acute lupus pneumonitis, diffuse alveolar hemorrhage, pulmonary embolism (usually associated with antiphospholipid syndrome), pulmonary hypertension, vasculitis, shrinking lung syndrome, pulmonary nodules, bronchiolitis obliterans, infections, and diaphragmatic weakness [1, 4, 5]. The most common is pleuritis, which can be associated with pleural effusions [4]. Diffuse alveolar hemorrhage is a rare but potentially catastrophic manifestation with high mortality. Although DAH is known to complicate collagen vascular disorders, it is a rare occurrence [1, 6]. Among rheumatologic diseases, it occurs most frequently in patients with SLE and systemic vasculitis [7]. Active lupus nephritis with hypoalbuminemia is a major risk factor. Alveolar hemorrhage may occur despite ongoing treatment with corticosteroids and immunosuppressive therapy. Patients with poorly controlled disease can have recurrent episodes of DAH [1].

## 2. Case Report

A 23-year-old Hispanic male presented with 5 days of shortness of breath and chest pain. Chest pain was retrosternal 8/10, stabbing, constant, positional, reproducible on deep inspiration, radiating to left side of chest and left shoulder, and relieved somewhat by sitting forward and upright. The patient also reported worsening of his body aches for the same duration. Patient's medical history was significant for SLE diagnosed 6 years ago, Henoch-Schonlein purpura, class III nephritis, anemia, antiphospholipid syndrome, and rheumatoid arthritis and he had not been taking his medications (prednisone, hydroxychloroquine, and mycophenolate mofetil) for two years due to the side effects of weight gain and depression. 

**Figure 1:**
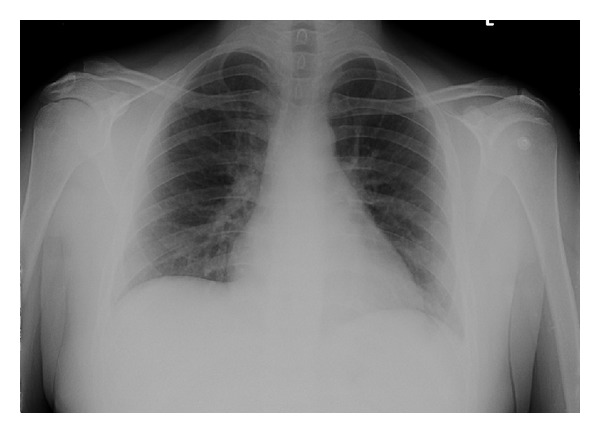
Patient's Chest radiograph on admission with no infiltrates.

Physical exam was noteworthy of symmetric swelling of upper and lower extremities with multiple ecchymosis and petechiae, joint tenderness without evidence of synovitis, and clear lungs with no rhonchi/wheeze/crackles. No pleural or pericardial rub was noted. On admission, the patient's hemoglobin was 10.4 g/dL, hematocrit 29.5%, white cell count 3100 cell/mm^3^, BUN 40 mg/dL, creatinine 1.6 mg/dL, and urine protein excretion 2.4 g/day. Labs revealed elevated double-stranded DNA titer at >320 with low complements; C3 was 31 mg/dL (standard reference value 50–180 mg/dL), and C4 was 3 mg/dL (standard reference value 10–40 mg/dL). Rest of the laboratory work-up was within normal limits. Chest radiograph showed slightly increased interstitial markings without any infiltrates (Figure 1). Intravenous methylprednisolone 20 mg every eight hours and hydroxychloroquine 200 mg twice daily were initiated for SLE flare.

**Figure 2:**
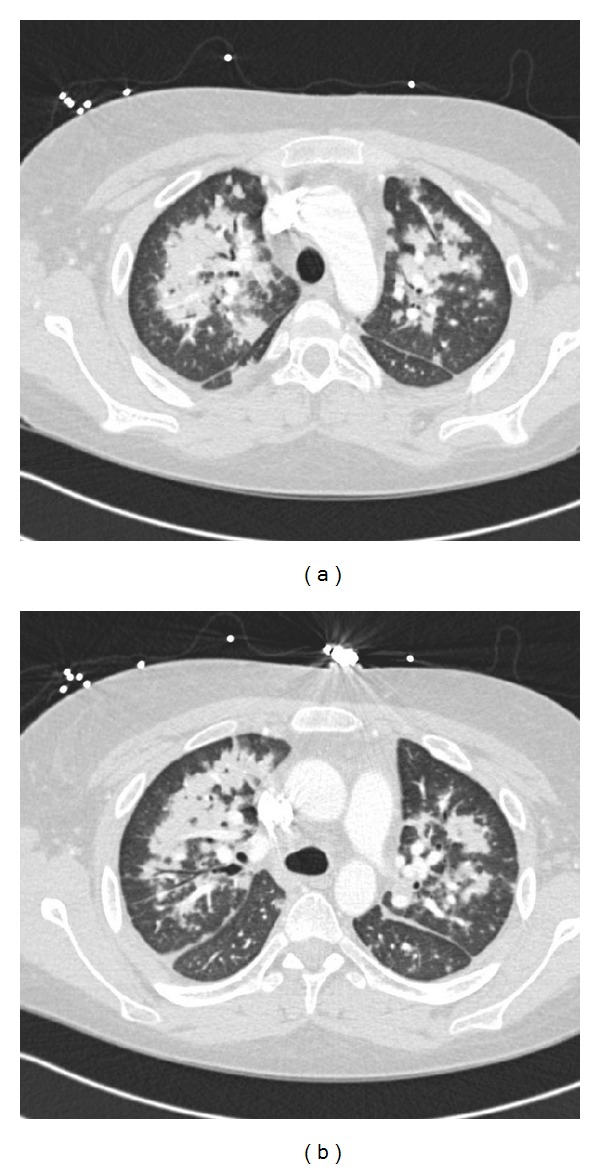
CT Thorax obtained after patient developed hemoptysis and dyspnea showing bilateral alveolar infiltrates.

The patient had improvement in his symptoms with the above treatment, but on day 5 of hospital admission, he had temperature of 38.2 C along with multiple episodes of hemoptysis accompanied with oxygen desaturation which got progressively worse. CT thorax with contrast revealed diffuse alveolar opacities in a central, perihilar distribution (Figure 2). The patient was intubated and placed on mechanical ventilation, and fiberoptic bronchoscopy was performed. Alveolar hemorrhage was confirmed as a source of hemorrhage; specimens for bronchoalveolar lavage, AFB cultures, and cytology were obtained. Methylprednisolone was increased to 500 mg daily; broad spectrum antibiotic coverage was initiated until culture results were obtained. One dose of intravenous cyclophosphamide 700 mg was given.

The patient remained afebrile, hemodynamically stable and was successfully weaned off ventilator support after 3 days. No further episodes of hemoptysis occurred; his hemoglobin remained stable around 8.4 g/dL and did not require blood or blood product transfusions. Patient's creatinine improved to 0.8 mg/dL. Cultures from blood, sputum, and bronchoalveolar lavage remained negative, so antibiotics were deescalated. Chest X-ray showed resolving infiltrates. Kidney biopsy revealed class IV lupus nephritis. The patient was counseled about his disease, stressed compliance and was discharged from medicine floor with followup in clinics.

## 3. Discussion

SLE is considered primarily a disease of child-bearing women; typical age at diagnosis is between 15 and 45 years. The female-to-male ratio for SLE is 9 : 1, while for development of DAH in an SLE female-to-male ratio is approximately 6 : 1 [5]. DAH remains rare but a life-threatening complication in SLE. It is an uncommon complication with estimates ranging from <2 to 5.4% in cohorts of lupus patients and accounts for 1.5 to 3.7% of hospital admission due to SLE [4, 7–9]. The mean age of onset in DAH is around 30 years. DAH tends to occur early in the disease; in most studies, mean duration of SLE ranged between 1.8 and 4.5 years before its first episode [1, 8, 9]. In a case series mean duration of DAH was 7 days with range between 2 and 21 days [1]. DAH usually occurs in patients with established diagnosis of SLE; however, cases have been reported where DAH was initial presentation of SLE [10, 11].

There is marked variability in survival over different case series reports, with reported mortality rates of 23 to 92%. Mortality rates for time intervals 1980–1989, 1990–1999, and 2000–2009 were 81.2%, 57.2%, and 35.1%, respectively [4]. Improvement in survival could be related to earlier diagnosis, aggressive treatment, use of broad spectrum antibiotics, supportive measures, and better management of ventilator support.

Since DAH is a rare complication and very few studies have been published, pathogenic mechanisms of DAH in SLE are not completely understood. The proposed mechanisms include immune-mediated damage of small blood vessels and alveolar space. Histopathologically three different patterns have been described: bland pulmonary hemorrhage, pulmonary capillaritis, and diffuse alveolar damage [5, 7, 12–16]. Most cases are associated with bland hemorrhage without any changes of vasculitis, interstitial inflammation, or infection; however, few recent series have shown increased incidence of pulmonary capillaritis [1, 14, 17, 18].

Patients presenting with “classic triad” of hemoptysis, rapid fall in hemoglobin over 24–48 hours, and new alveolar or interstitial infiltrates should have high suspicion for DAH. Hemoptysis is however variable and is not reliable, present initially in only half of the cases [1, 4, 7]. Fever, dyspnea, cough, chest pain, and pleural effusion are also commonly associated with DAH. Most common extrapulmonary manifestation is renal involvement, usually class III or IV nephritis [1, 7]. Moreover, an increased risk for DAH has been reported with active renal disease, especially when manifesting as nephrotic syndrome [4]. Serologically high titers of anti-dsDNA, hypocomplementemia, and anemia are frequently noted [1, 7, 9]. Hypoxemia of varying degrees results from ventilation-perfusion abnormalities produced by DAH. Serial measurements of diffusing capacity for carbon monoxide (DLCO) have been suggested as sensitive marker for active alveolar bleeding [7]. Radiologically acute diffuse alveolar infiltrates, usually perihilar or basilar with sparing of apices and periphery, are noted [1, 7–10, 17–19]. High-resolution chest radiography has a limited use but has been suggested to be superior to chest radiographs [20]. If DAH is suspected, bronchoscopy usually confirms the diagnosis; sequential bronchoalveolar lavage sample from the same location with increasing red blood cell count is regarded as diagnostic [21]. In subacute cases, methods such as quantitative scoring of the hemosiderin-laden macrophages from BAL fluid or increasing red blood cell counts in sequential aliquots of BAL fluid may be useful. Hemosiderin, a product of hemoglobin degradation, appears at least 48 hours after bleeding [22, 23].

As prospective controlled studies are lacking, so optimal management therapy of DAH has not been established. Corticosteroids are the mainstay of therapy. The use of oral prednisone therapy in past was associated with high mortality. This prompted the introduction of high-dose intravenous pulse corticosteroids, but due to lack of data, there are no guidelines for the optimal dose and duration of therapy. The standard dose of “pulse” methylprednisolone as initial therapy is 1 g daily for 3 days, decreased to 1-2 mg/kg/day by fourth day, and switched to oral prednisone over next few days [7–9]. Additional adjuvant immunosuppressive agents can be started concurrently in critically ill patients or when there is lack of response to steroids. Use of cyclophosphamide has been linked to better survival as in our case [17, 19]. However, few case series report higher mortality when cyclophosphamide was used. In some studies, combination of pulse methylprednisolone and cyclophosphamide was associated with increased survival rates [17, 24]. Plasmapheresis use in SLE-associated alveolar hemorrhage has not been proven to be as effective as other autoimmune causes such as Goodpasture's syndrome and some vasculitis-related pulmonary bleeding. Even though it did not improve survival in study by Zamora et al., plasmapheresis has been considered effective in numerous cases and can be added when there is inadequate response to high-dose steroids and cyclophosphamide [25–27]. 

Supportive measure must be established early in the management. Timely antibiotics, close hemodynamic monitoring, blood product administration, and mechanical ventilation should be used when indicated [7, 9, 28, 29].

B-cell depletion therapy has shown promising results in management of SLE [24, 30–32]. Current available data supporting its use in DAH is limited and should be explored. One published case report in Germany documents response to rituximab therapy after patient had relapse of DAH within 10 days of 1st episode; case of successful treatment when used in initial combination therapy has been reported [18, 33], while a case series looking at use in SLE reported fatalities in 3 patients with DAH in whom rituximab was used [34]. In two different series of eight patients, pulmonary administration of human recombinant-activated factor VIIa was effective in stopping bleeding in alveolar hemorrhage and decreasing oxygen requirement [35, 36]. None of the patients in the series had SLE-related hemorrhage, but this option can be considered to achieve hemostasis when other therapies have failed.

The patient in our case presented with relatively common complaints and had rapid clinical deterioration. There is a large spectrum of pulmonary manifestations which can occur in SLE. This case emphasizes even though DAH is a rare complication of SLE, it should be considered in differential diagnosis of a patient with SLE presenting with any pulmonary complaint. This is essential as DAH has to be differentiated from CAPS (catastrophic antiphospholipid [Asherson's] syndrome) because treatment is different, and early aggressive management leads to a favorable outcome. Along with established therapies, use of B-cell depletion therapy, factor VIIa, and benefits of plasmapheresis/plasma exchange have shown promise and should be looked at in the future.
